# Transport of Pregabalin Via L-Type Amino Acid Transporter 1 (SLC7A5) in Human Brain Capillary Endothelial Cell Line

**DOI:** 10.1007/s11095-018-2532-0

**Published:** 2018-10-29

**Authors:** Yu Takahashi, Tomohiro Nishimura, Kei Higuchi, Saki Noguchi, Yuma Tega, Toshiki Kurosawa, Yoshiharu Deguchi, Masatoshi Tomi

**Affiliations:** 1 Division of Pharmaceutics, Faculty of Pharmacy, Keio University, 1-5-30, Shibakoen, Minato-ku, Tokyo, 108-8512 Japan; 2 Laboratory of Drug Disposition & Pharmacokinetics, Faculty of Pharma-Sciences, Teikyo University, 2-11-1, Kaga Itabashi-ku, Tokyo, 173-8605 Japan

**Keywords:** anti-epileptic drug, blood-brain barrier, L-type amino acid transporter, pregabalin

## Abstract

**Purpose:**

The anti-epileptic drug pregabalin crosses the blood-brain barrier (BBB) in spite of its low lipophilicity. This study was performed to determine whether L-type amino acid transporters (LAT1/SLC7A5 and LAT2/SLC7A8) contribute to the uptake of pregabalin.

**Methods:**

Pregabalin uptake by LATs-transfected HEK293 cells or hCMEC/D3 cells, an *in vitro* human BBB model, was measured by LC-MS/MS analysis. Expression of LAT1 mRNA in hCMEC/D3 cells was determined by quantitative RT-PCR analysis.

**Results:**

Overexpression of LAT1, but not LAT2, in HEK293 cells significantly increased the cellular uptake of pregabalin, and the LAT1-mediated uptake was saturable with a *K*_m_ of 0.288 mM. LAT1-mediated amino acid uptake was inhibited specifically and almost completely in the presence of 1 mM pregabalin. The uptake of pregabalin by hCMEC/D3 cells was sodium-independent, saturable (*K*_m_ = 0.854 mM), and strongly inhibited by large amino acids at 1 mM, 2-aminobicyclo-(2,2,1)-heptane-2-carboxylic acid, a specific system L inhibitor, at 1 mM and by JPH203, a LAT1-selective inhibitor, at 10 μM. Pregabalin uptake in hCMEC/D3 cells was also decreased by 75% by the silencing of LAT1 gene using LAT1 siRNA.

**Conclusions:**

Our results indicate that LAT1, but not LAT2, recognizes pregabalin as a substrate. It is suggested that LAT1 mediates pregabalin transport at the BBB.

**Electronic supplementary material:**

The online version of this article (10.1007/s11095-018-2532-0) contains supplementary material, which is available to authorized users.

## Introduction

Pregabalin, a ligand of the α_2_δ subunit of voltage-gated calcium channels, is used to treat epilepsy, as well as neuropathic pain and fibromyalgia. It reduces the influx of calcium ions into neurons and the subsequent release of neurotransmitters (1), and consequently has anticonvulsant and analgesic effects. The paracellular distribution of drugs from the circulating blood to the brain is strictly restricted by the blood-brain barrier (BBB). Nevertheless, pregabalin crosses the BBB with a brain extracellular fluid/plasma concentration ratio of 0.10 in rat (2), despite its low lipophilicity (log D at pH 7.4 of −1.35), and is thus pharmacologically active in the central nervous system. Hence, it appears that the BBB is equipped with an active carrier(s) mediating the brain distribution of pregabalin.

Pregabalin was originally designed as an analogue of the neurotransmitter γ-aminobutyric acid (GABA). Nevertheless, a previous study has demonstrated that 25 mM pregabalin had no effect on GABA uptake by cells overexpressing GABA transporters (GAT1/SLC6A1, GAT3/SLC6A11, and GAT2/SLC6A13) (3), and thus pregabalin seems not to interact with GATs. In addition, the permeability of GABA across the BBB was similar to that of typical paracellular markers such as sucrose, mannitol, and inulin (4), suggesting negligible activity of GABA transporters at the BBB. On the other hand, it has been reported that the uptake of pregabalin by Chinese hamster ovary and human colon adenocarcinoma Caco-2 cells is mutually exclusive with respect to L-leucine, gabapentin, and 2-aminobicyclo-(2,2,1)-heptane-2-carboxylic acid (BCH), which are substrates of amino acid transport system L (3). Therefore, system L may mediate pregabalin uptake in mammalian cells. So far, four subtypes of system L with different substrate selectivity and transport properties have been described: LAT1/SLC7A5, LAT2/SLC7A8, LAT3/SLC43A1, and LAT4/SLC43A2 (5–7). However, the subtype(s) responsible for pregabalin uptake has not been identified yet.

LAT1 and LAT2 are functionally expressed in the brain, whereas the expression levels of *Lat3* and *Lat4* mRNAs in the brain are minimal (6,7). LAT1 and LAT2 work as exchangers for large neutral amino acids, such as L-leucine, L-phenylalanine, L-histidine, and L-tryptophan, and also thyroid hormones, including triiodothyronine (T_3_) and thyroxine (T_4_), in a sodium-independent manner (8,9). LAT1 also recognizes some drugs, such as L-dopa and gabapentin (10,11), while LAT2 accepts small neutral amino acids (12). LAT1 is located at the luminal and abluminal membranes of rodent brain capillary endothelial cells forming the BBB (13,14). Quantitative proteomics showed that LAT1 protein expression in human brain capillaries amounts to 0.43 fmol/μg-protein (15). Accordingly, LAT1 at the BBB is considered to mediate the brain distribution of L-dopa and gabapentin. As for LAT2, although its mRNA is expressed in rat primary cultured brain capillary endothelial cells (16), its functional activity is likely to be minimal since it was reported that the brain uptake of L-leucine was not inhibited by small neutral amino acids in rat brain perfusion studies (17). Instead, LAT2 is expressed in rat primary cultured astrocytes and neurons (18,19). The neuronal localization of *Lat2* mRNA was confirmed by *in-situ* hybridization in mouse brain sections (20). Accordingly, LAT1 and LAT2 are candidates for transporting pregabalin across the BBB and for regulating intracerebral pregabalin distribution, respectively.

In the present study, we examined the plasma membrane transport mechanism of pregabalin at the BBB using immortalized human brain capillary endothelial (hCMEC/D3) cells as a well-established *in-vitro* model of human brain microvascular endothelial cells (21). In hCMEC/D3 cells, although the expression level of LAT1 protein is below of the quantification limit on quantitative proteomics (22), *LAT1* and *LAT2* mRNAs are known to be expressed (23). The functional expression of LAT1 in hCMEC/D3 cells has been also confirmed using a LAT1-targeting siRNA, which evoked a reduction of gabapentin uptake (10).

## Materials and Methods

### Chemicals

L-Leucine, [^14^C] ([^14^C]L-leucine: 329 mCi/mmol) was purchased from PerkinElmer (Boston, MA). Pregabalin was obtained from Toronto Research Chemicals (Ontario, Canada); 2-aminobicyclo-(2,2,1)-heptane-2-carboxylic acid (BCH) and α-methylaminoisobutyric acid (MeAIB) were from Sigma-Aldrich (St.Louis, MO); JPH203 was from Chemscene (Monmouth Junction, NJ); gabapentin, unlabeled L-amino acids, *p*-aminohippuric acid (PAH), and tetraethylammonium (TEA) were from Wako (Osaka, Japan).

### Uptake Studies by LATs-Overexpressing Cells

HEK293 cells were maintained in Dulbecco’s modified Eagle’s medium (Nacalai tesque, Kyoto, Japan) supplemented with 10% fetal bovine serum (FBS; Thermo Scientific, Waltham, MA), 2 mM L-glutamate, 100 U/mL benzylpenicillin, and 100 mg/mL streptomycin at 37°C in a humidified atmosphere of 5% CO_2_ in air. HEK293 cells were transiently transfected with pIRES2-dsRed mammalian expression vector (0.40 μg/cm^2^) containing the entire coding region for human *SLC7A5* or *SLC7A8* cDNA, which encodes LAT1 or LAT2, respectively, or with the vector alone (mock), by using Lipofectamine 2000 (Life Technologies, Carlsbad, CA), and then cultured for 48 h.

The uptake experiment was performed using a silicone layer filtering centrifugation technique as reported (24,25). Briefly, the cells were scraped off dishes and suspended in extracellular fluid (ECF) buffer (122 mM NaCl, 25 mM NaHCO_3_, 3 mM KCl, 1.4 mM CaCl_2_, 2 mM MgSO_4_, 0.4 mM K_2_HPO_4_, 10 mM D-glucose, 10 mM HEPES, and pH 7.4). The cell suspension was mixed with ECF buffer containing pregabalin or [^14^C]L-leucine, and then incubated at 37°C. At a designated time, cells were separated from the buffer by centrifugal filtration at 10,000×g for 2 min through a silicone oil layer (density of 1.03) placed over a 3 N KOH solution. The cell lysate in KOH was then neutralized with HCl.

### Uptake Studies by hCMEC/D3 Cells

hCMEC/D3 is a human brain capillary endothelial cell line immortalized by lentiviral transduction of the catalytic subunit of human telomerase and SV40-T antigen (21). hCMEC/D3 cells were maintained in EBM-2 medium (Lonza, Basel, Switzerland) supplemented with 2.5% FBS, vascular endothelial growth factor, insulin-like growth factor-1, epidermal growth factor, basic fibroblast growth factor, and hydrocortisone, penicillin, and streptomycin at 37°C in a humidified atmosphere of 5% CO_2_ in air. hCMEC/D3 cells between passages 25 and 35 were seeded on rat collagen I-coated 24-well plates (Corning, Corning, NY) at a density of 2.0 × 10^4^ cells/cm^2^. The uptake was evaluated after the cells reached confluence.

Uptake experiments were conducted as described previously (26), with some modifications. Briefly, the medium was removed and the cells were washed twice and preincubated with ECF buffer at 37°C for 10 min. To obtain a sodium-free condition, sodium in the ECF buffer was substituted with equimolar choline. After preincubation, uptake was initiated by replacing the preincubation buffer with uptake buffer (ECF buffer or sodium-free ECF buffer) containing prebagalin in the absence or presence of inhibitors. The uptake was terminated by removal of the uptake buffer and the cells were immediately washed three times with ice-cold buffer, and then solubilized overnight in 0.1 N NaOH at room temperature. Aliquots were neutralized with 0.1 N HCl.

### Quantification of Pregabalin and [^14^C]L-Leucine Uptake

Pregabalin was quantitated by tandem mass spectrometry (LCMS-8050; Shimadzu, Kyoto, Japan) coupled to high-performance liquid chromatography (Shimadzu) (LC-MS/MS). Samples were deproteinized with the same volume of acetonitrile containing 100 nM gabapentin (internal standard), and an aliquot (4 μL) was injected into the LC-MS/MS. Mobile phases A and B consisted of water and acetonitrile, respectively, and both contained 0.1% formic acid and 2 mM ammonium acetate. Chromatographic separation was performed on a Capcell Pak C18 UG120 column (2.0 mm i.d. × 150 mm, 5 μm; Shiseido, Tokyo, Japan) at 40°C with a gradient of mobile phase B: 5% for 4.3 min (at 0 to 4.3 min), 5% to 100% for 2.2 min (at 4.3 to 6.5 min), 100% for 2.5 min (at 6.5 min to 9 min), and 5% for 3 min (at 9 to 12 min); the flow rate was 0.3 mL/min. Mass spectrometric detection was performed by multiple reaction monitoring in the electrospray ionization positive ion mode, using m/z 160.2 → 55.1 for pregabalin and m/z 172.2 → 154.2 for gabapentin. Radioactivity of [^14^C]L-leucine was measured with a liquid scintillation counter. Cellular protein amount was determined by using a bicinchoninic acid (BCA) Protein Assay Kit (Pierce, Rockford, IL), with bovine serum albumin (BSA) as a standard.

Uptake of substrates was expressed as cell-to-medium ratio, indicating the concentration ratio (μL/mg protein) of pmol/mg protein in the cells to pmol/μL in the medium. The LAT-mediated uptake was calculated by subtracting the uptake in mock-transfected cells from that in LAT-overexpressing cells.

### Kinetic Analysis

The kinetic parameters of pregabalin uptake via LAT1 and in hCMEC/D3cells were estimated by fitting to the following *equation1* and *equation2*, respectively, by means of nonlinear least-squares regression analysis using GraphPad Prism 4.0 (GraphPad Software Inc., La Jolla, CA).

1 $$ V=\left({V}_{max}\times \left[S\right]\right)/\left({K}_m+\left[S\right]\right) $$

2 $$ V=\left({V}_{max}\times \left[S\right]\right)/\left({K}_m+\left[S\right]\right)+{K}_d\times \left[S\right] $$

*V*, [S], *K*_m_, *V*_max_, and *K*_d_ represent uptake velocity of pregabalin uptake, concentration of pregabalin, Michaelis-Menten constant, maximum uptake velocity, and nonsaturable uptake clearance, respectively. All kinetic parameters were expressed as the mean ± SD.

### RNA Interference Analysis

Gene silencing of LAT1 was performed as described previously (27), with some modifications. Briefly, hCMEC/D3 cells were seeded onto 24-well plates, and siRNA transfection was performed with lipofectamine RNAiMAX (Invitrogen). For gene silencing of LAT1, we used two sets of LAT1 siRNAs (Silencer® Select: s15653 and s15655, Thermo Scientific) with a final concentration of each 5 nM. At 72 h after transfection, the cells were used for RNA extraction or cellular uptake studies.

### RNA Extraction and Quantitative RT-PCR

Total RNA was extracted from hCMEC/D3 cells using RNeasy Mini Kit (QIAGEN, Valencia, CA). First-strand cDNA was synthesized from the isolated total RNA as templates with High-Capacity RNA-to-cDNA™ Kit (Thermo Scientific), and then used for quantitative RT-PCR analysis which performed by CFX Connect™ real time PCR detection system (Bio-Rad, Hercules, CA) with Power SYBR® Green Master Mix (Thermo Scientific) according to the manufacturer’s instructions. Specific primer sets for quantitative RT-PCR analysis were designed as follows: 5′- GCA TCG GCT TCA CCA TCA TC -3′ as forward and 5′- ACC ACC TGC ATG AGC TTC TGA C -3′ as reverse for LAT1, and 5′- GCG AGA AGA TGA CCC AGA TC -3′ as forward and 5′- CCA GTG GTA CGG CCA GAG G -3′ as reverse for β-actin. The relative mRNA expression levels of LAT1 was calculated by the ΔΔCt method for the relative quantification based on the mRNA level of β-actin, a housekeeping gene.

### Statistical Analysis

Statistical analyses were performed by the use of an unpaired, two-tailed Student’s *t* test for comparisons between two groups, or one-way ANOVA followed by Dunnett’s post hoc test for multiple comparisons. The value of *p* < 0.05 was taken as the criterion for a statistically significant difference.

## Results

### Uptake of Pregabalin in LAT-Overexpressing HEK293 Cells

**Fig. 1:**
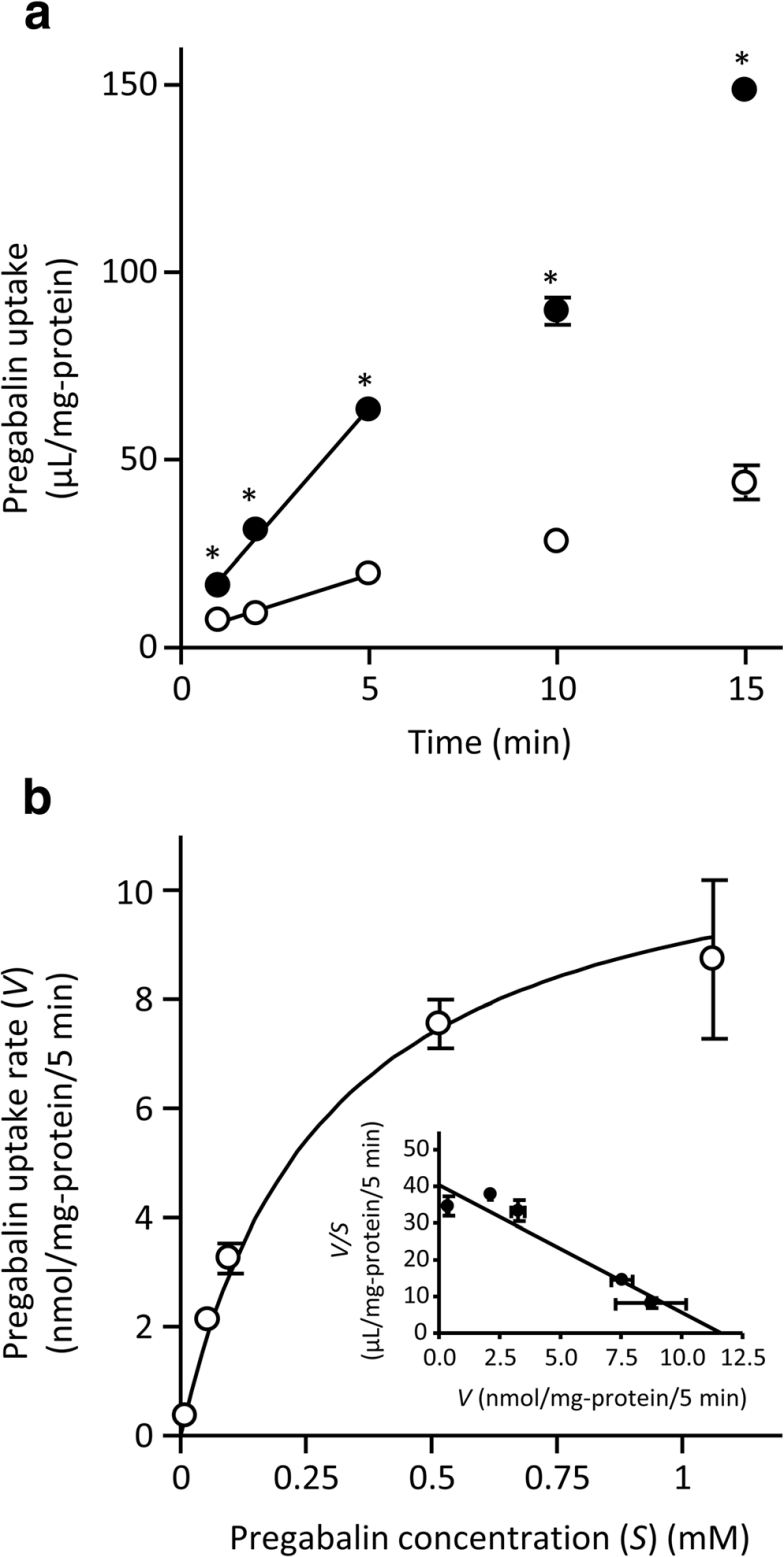
**Time course (a) and concentration dependence (b) of LAT1-mediated pregabalin uptake.** (a) Pregabalin uptake (10 μM) by LAT1-overexpressing cells (●) and mock cells (○) was examined at 37°C. *p < 0.05, significantly different from mock cells. (b) Pregabalin uptake by LAT1-overexpressing cells and mock cells was examined at 37°C for 5 min. Data represent LAT1-mediated uptake calculated by subtracting the uptake in mock cells from that in LAT1-overexpressing cells. Data were subjected to Michaelis–Menten (main) and Eadie–Scatchard (inset) analyses. Each point represents the mean ± SEM (*n* = 3).

First, we confirmed that the uptake of [^14^C]L-leucine (0.4 μM) by HEK293 cells overexpressing LAT1 or LAT2 was significantly higher than that by mock cells. LAT1 and LAT2 require 4F2 heavy chain (4F2hc) for their functional expressions by forming a heterodimeric complex. It is expected that exogenously transfected LAT1 or LAT2 was functionally expressed in the plasma membrane as heterodimeric complexes with endogenous 4F2hc (Table I). In order to clarify whether LAT1 and LAT2 accept pregabalin as a substrate, cellular uptake studies of 10 μM pregabalin were conducted. Only LAT1-overexpressing cells showed significantly higher pregabalin uptake compared to mock cells (Table I). The initial uptake rate of pregabalin over 5 min in LAT1-overexpressing cells was 11.5 μL/mg-protein/min, which was 3.60-fold greater than that in mock cells (3.20 μL/mg-protein/min) (Fig. 1a). LAT1-mediated pregabalin uptake was saturable with a *K*_m_ of 0.288 ± 0.049 mM and a *V*_max_ of 11.6 ± 1.4 nmol/mg-protein/5 min (Fig. 1b). We also evaluated the inhibitory effect of pregabalin on [^14^C]L-leucine uptake. Pregabalin at 1 mM almost completely blocked the LAT1-mediated uptake of [^14^C]L-leucine, whereas it had no effect on the uptake mediated by LAT2 (Table II). These results indicate that, of the two LAT subtypes, only LAT1 specifically recognized pregabalin.

**Table I:** LAT-mediated uptake of [^14^C]L-leucine and pregabalin

	[^14^C]L-Leucine uptake (μL/mg-protein)	Pregabalin uptake (μL/mg-protein)
Mock	247 ± 5	22.2 ± 1.9
LAT1	485 ± 29*	57.2 ± 1.7*
LAT2	345 ± 13*	21.4 ± 0.9

LAT1-, LAT2-overexpressing, and mock cells were incubated with [^14^C]L-leucine (0.4 μM) or pregabalin (10 μM) at 37°C for 2 or 5 min, respectively. Each value represents the mean ± SEM (n = 3). *p < 0.05, significantly different from mock cells

**Table II:** Inhibitory effect of pregabalin on LAT1 and LAT2-mediated amino acid uptake

	[^14^C]L-Leucine uptake (μL/mg-protein)		Transporter-mediated [^14^C]L-leucine uptake (μL/mg-protein)	
	Control	+ 1 mM pregabalin	Control	+ 1 mM pregabalin
Mock	247 ± 5	66.4 ± 4.5*	–	–
LAT1	485 ± 29	103 ± 7*	237 ± 29 [100]	36.8 ± 8.5* [15.5]
LAT2	345 ± 13	192 ± 7*	97.8 ± 14 [100]	126 ± 6 [129]

LAT1-, LAT2-overexpressing, and mock cells were incubated with [^14^C]L-leucine (0.4 μM) in the presence or absence (Control) of 1 mM pregabalin at 37°C for 2 min. Transporter-mediated uptake was calculated by subtracting the uptake by mock cells from that by LAT-overexpressing cells. Values in square brackets represent percentage of control uptake. Each value represents the mean ± SEM (n = 3). *p < 0.05, significantly different from control

### Characteristics of Pregabalin Uptake by hCMEC/D3 Cells

**Fig. 2:**
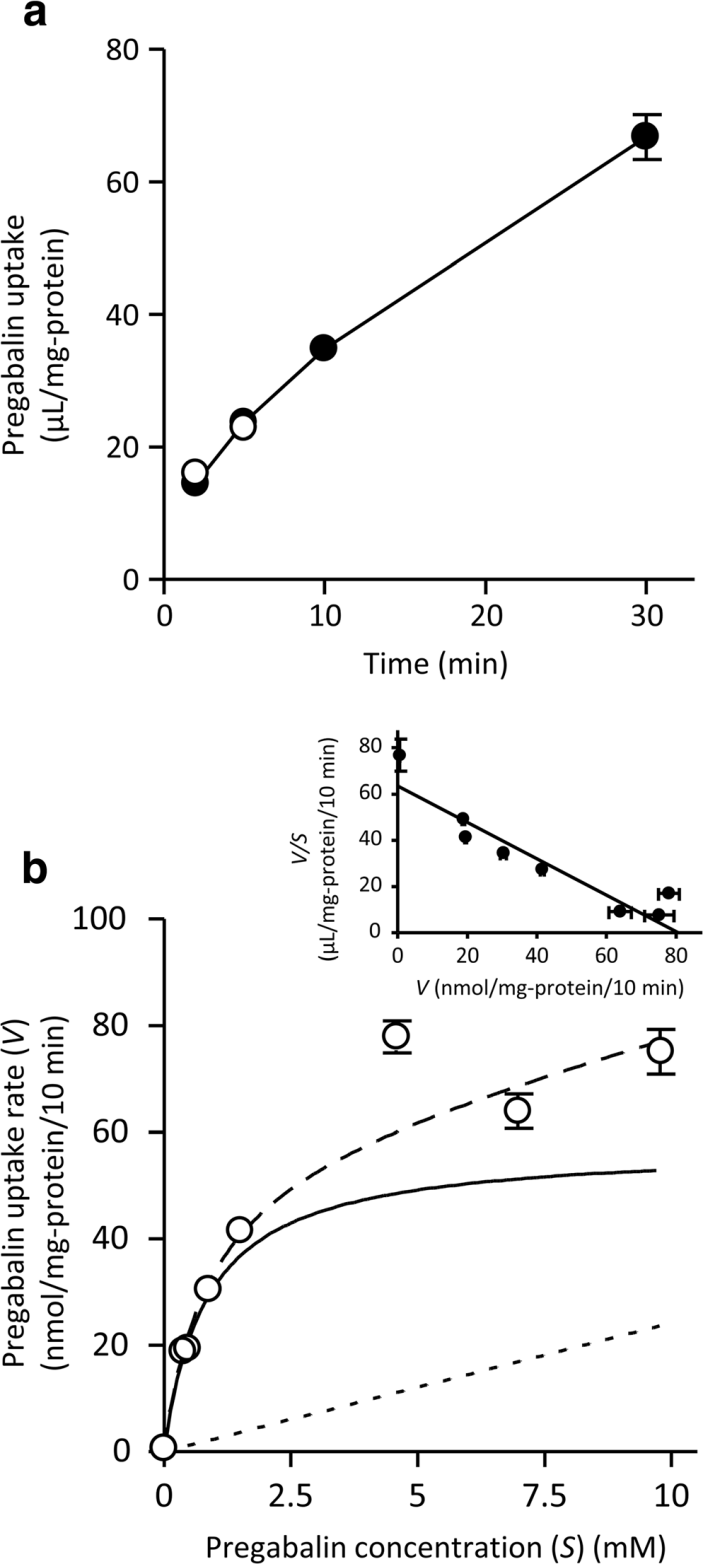
**Time course (a) and concentration dependence (b) of pregabalin uptake by hCMEC/D3 cells.** (a) Pregabalin uptake (10 μM) by hCMEC/D3 cells was examined at 37°C in the presence (●) or absence (○) of sodium. (b) Pregabalin uptake by hCMEC/D3 cells was examined at 37°C for 10 min. Data were subjected to Michaelis–Menten (main) and Eadie–Scatchard (inset) analyses. In the Michaelis–Menten plot, the solid, dotted, and dashed lines represent saturable, nonsaturable, and overall pregabalin uptake, respectively. In the Eadie–Scatchard plot, the solid line represents the overall pregabalin uptake. Each point represents the mean ± SEM (n = 3–4).

The uptake of pregabalin (10 μM) by hCMEC/D3 cells, an *in vitro* human BBB model, gradually increased over 30 min (Fig. 2a). The uptake over 2–5 min was almost the same as that under sodium-free conditions (Fig. 2a), indicating that pregabalin uptake by hCMEC/D3 cells is sodium-independent. Moreover, the uptake was saturable with a *K*_m_ of 0.854 ± 0.287 mM and a *V*_max_ of 58.1 ± 15.2 nmol/mg-protein/10 min, and a *K*_d_ of 2.43 ± 1.80 μL/mg-protein/10 min (Fig. 2b). As shown in Table III, pregabalin uptake by hCMEC/D3 cells was almost completely inhibited by typical LAT1 substrates and inhibitors, such as L-leucine, L-phenylalanine, L-histidine, L-tryptophan, and 2-aminobicyclo-(2,2,1)-heptane-2-carboxylic acid (BCH) at 1 mM, and also by JPH203, a LAT1 selective inhibitor (28), at 10 μM. On the contrary, the inhibitory effect of α-methylaminoisobutyric acid (MeAIB), a specific inhibitor for the amino acid transport system A, at 10 mM on pregabalin uptake was weak, and L-glutamate and L-arginine, both at 1 mM, were not inhibitory. Tetraethylammonium (TEA) and *p*-aminohippuric acid (PAH), inhibitors for organic cation and anion transporters, respectively, both at 1 mM, neither affected to the uptake. These results suggest that LAT1 predominantly contributes to pregabalin transfer in hCMEC/D3 cells.

**Table III:** Inhibitory effect of various compounds on pregabalin uptake by hCMEC/D3 cells

	Compound	Uptake (% of Control)
	Control	100 ± 5
	Na^+^ − free	102 ± 7
1 mM	BCH	17.0 ± 3.0*
1 mM	L-Leu	16.7 ± 2.7*
1 mM	L-His	20.6 ± 1.0*
1 mM	L-Phe	ULQ (< 6.15)
1 mM	L-Trp	ULQ (< 5.66)
10 μM	JPH203	ULQ (< 6.53)
1 mM	L-Glu	117 ± 6
1 mM	L-Arg	101 ± 2
10 mM	MeAIB	80.8 ± 4.0*
1 mM	TEA	89.8 ± 4.3
1 mM	PAH	115 ± 7

hCMEC/D3 cells were incubated with pregabalin (10 μM) in the presence or absence (Control) of various compounds at 37°C for 10 min. Each value represents the mean ± SEM (*n* = 4–12). The uptake in the presence of 1 mM L-Phe, 1 mM L-Trp, or 10 μM JPH203 was below lower limit of quantification. ULQ means under the limit of quantification. The value in parentheses following ‘ULQ’ represents the value of the quantification limit. *p < 0.05, significantly different from control

### Effect of LAT1 siRNA Transfection on hCMEC/D3 Cells

**Fig. 3:**
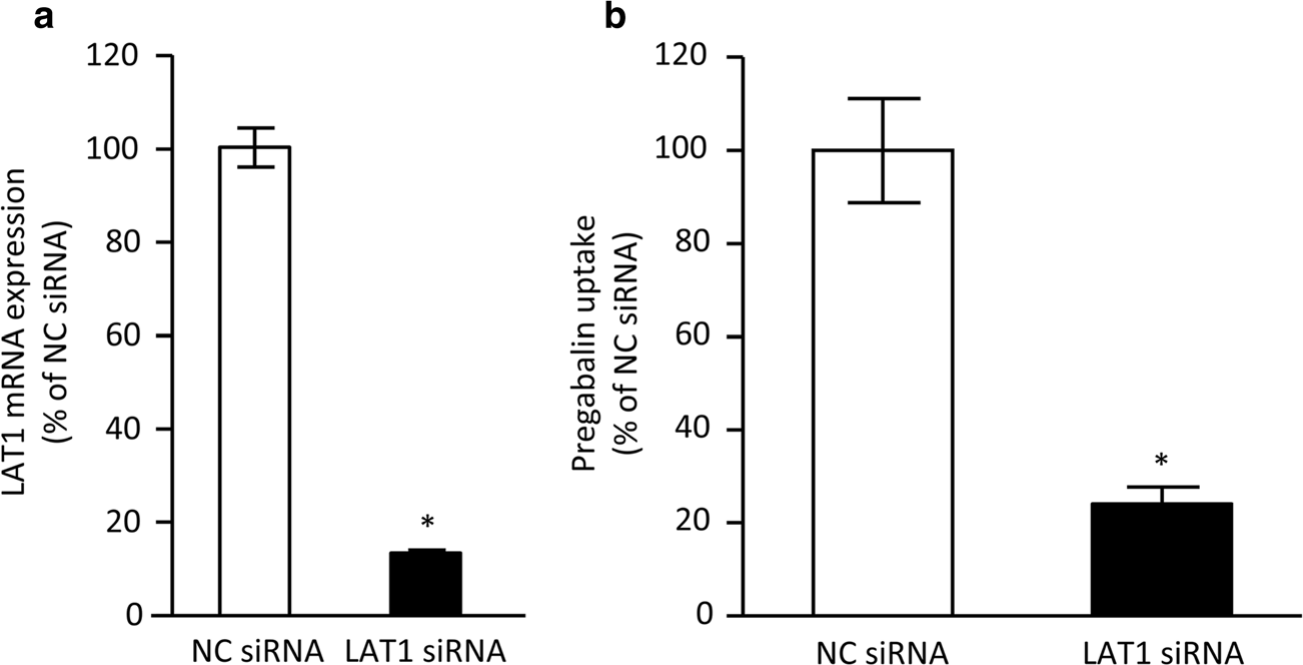
**Effect of LAT1 siRNA on the expression of LAT1 mRNA (a) and pregabalin uptake (b) in hCMEC/D3 cells.** (a) hCMEC/D3 cells were treated with negative control siRNA (NC siRNA) or LAT1 siRNA. (b) At 72 h after transfection of NC siRNA or LAT1 siRNA, pregabalin uptake (10 μM) by hCMEC/D3 cells was examined at 37°C for 10 min. Each column represents the mean ± SEM (n = 3–4). *p < 0.05, significantly different from NC siRNA.

As we further elucidate the contribution of LAT1 to pregabalin uptake in hCMEC/D3 cells, we have observed the effect of LAT1 knockdown on the uptake. By the transfection of LAT1 siRNA, the expression of LAT1 mRNA in hCMEC/D3 cells was decreased more than 80% compared to negative control (Fig. 3a). This silencing of LAT1 gene in hCMEC/D3 cells decreased the cellular uptake of pregabalin (10 μM) by 75% (Fig. 3b), suggesting that LAT1 is responsible for pregabalin uptake in hCMEC/D3 cells. This result was in good agreement with that of inhibition by JPH203 shown in Table III.

## Discussion

In this study, we have firstly shown that LAT1 works as a pregabalin transporter and is predominantly responsible for pregabalin uptake in hCMEC/D3 cells, an *in vitro* model of human brain microvascular endothelial cells. The involvement of LATs in the pregabalin uptake by hCMEC/D3 cells was supported by sodium independency and the potent inhibitory effects of large neutral amino acids and BCH on pregabalin uptake (Fig. 2a and Table III). Only LAT1-overexpressing cells showed uptake of pregabalin and inhibition of L-leucine uptake by 1 mM pregabalin, while LAT2-overexpressing cells showed neither (Tables I and II, and Fig. 1). We further examined the effects of JPH203, a LAT1-selective inhibitor, and LAT1 siRNA on pregabalin uptake by hCMEC/D3 cells. It has reported that JPH203 did not inhibit L-leucine uptake mediated by human LAT2 overexpressing cells (28). In addition, L-leucine uptake by LNCaP cells, a LAT3 and LAT4-domionant prostate cancer cell line, was not altered by treatment of JPH203, even though the uptake was significantly decreased by treatment of BCH (29), indicating that JPH203 has a high specificity for the inhibition of LAT1 among system L. We demonstrated the significant decreasing in the pregabalin uptake by hCMEC/D3 cells in either case of the treatment of JPH203 and LAT1 siRNA (Table III and Fig. 3b). These results strongly suggest that LAT1 plays a role in plasma membrane transport of pregabalin in human brain microvascular endothelial cells, as in the cases of the transport of L-dopa (13) and gabapentin (10).

Amino acid transport system A consists of 3 subtypes, sodium-coupled neutral amino acid transporter (SNAT) 1/SLC38A1, SNAT2/SLC38A2, and SNAT4/SLC38A4, and plays an important role in a transfer of small neutral amino acids, such as L-alanine, L-serine, and L-cysteine, in a sodium-dependent manner (30). The pregabalin uptake by hCMEC/D3 cells was slightly inhibited by 10 mM MeAIB, a specific inhibitor of system A (Table III). In contrast, the uptake did not show sodium dependency (Fig. 2a), ruling out participation of system A. This was confirmed by the absence of any significant difference of pregabalin uptake among SNAT1-, SNAT2-, or SNAT4-overexpressing cells and mock-transfected cells (Supplemental table1).

It has been reported that organic cation transporter novel type 1 (OCTN1/SLC22A4) is a mediator of the intestinal absorption of pregabalin (31) and is expressed in hCMEC/D3 cells, at least at the mRNA level (27). In order to evaluate the contribution of OCTN1 to the pregabalin uptake by hCMEC/D3 cells, we utilized TEA at 1 mM, which reportedly inhibited OCTN1-mediated TEA uptake by 50–80% (32,33). However, 1 mM TEA showed little inhibitory effect on the pregabalin uptake (Table III). This result is consistent with a previous report that uptake of gabapentin, the substrate of both LAT1 and OCTN1, in hCMEC/D3 cells was hardly affected by any OCTN inhibitors, such as verapamil, amantadine, and corticosterone (10). At the BBB, it was reported that the brain/plasma concentration ratio of intravenously administered ergothioneine, a typical substrate of OCTN1, was unchanged in Octn1-knockout mice (34), suggesting that functional OCTN1 may be negligible.

LAT2 is expressed in astrocytes and neurons, and has been proposed to be involved in homeostasis of amino acids and thyroid hormones (18,19). The negligible transport of pregabalin by LAT2 (Table I) implies that, once pregabalin is inside the brain parenchyma, its distribution to astrocytes and neurons via LAT2 may also be negligible. Instead, OCTN1 may mediate the transport of pregabalin into neurons, since OCTN1 is reportedly localized in brain neurons (35). Nakamichi *et al.* have demonstrated that uptake of ergothioneine, a substrate of OCTN1, was observed in primary cultured cortical neurons and Neuro2a cells, a model of undifferentiated neurons, and the uptake by Neuro2a cells was significantly decreased by suppression of OCTN1 (35).

In this study, the *K*_m_ values for pregabalin uptake via LAT1 and in hCMEC/D3 cells were estimated to be 0.288 mM and 0.854 mM, respectively (Fig. 1b and 2b). The reason for the difference in the obtained *K*_m_ values is not clear, but may be due to varying experimental conditions or phosphorylation levels in each cell type. It has been demonstrated that the affinity of the transporter was altered by its protein phosphorylation: *K*_m_ value of GABA transporter, serotonin transporter and glucose transporter was respectively modulated by Protein Kinase C, A, or Protein Phosphatase p38 mitogen-activated protein kinase (MAPK) signaling (36–38). Since the pregabalin plasma concentration in the clinical context (range of 1.8 to 89.2 μM) (39) is lower than the estimated *K*_m_, pregabalin influx via LAT1 at the BBB would not be saturated by pregabalin itself. In contrast, we estimated the transport activity of LAT1 by using the following equation, 1 / [1 + Σ(C_pl_ / *K*_m_)], based on the affinity of human LAT1-mediated amino acid transports (*K*_m_) (40) and the plasma concentration (C_pl_) of 8 LAT1 substrate amino acids (41); L-phenylalanine, L-leucine, L-isoleucine, L-tryptophan, L-tyrosine, L-histidine, L-valine, and L-methionine. The transport activity of LAT1 in the presence of the 8 amino acids at normal plasma concentrations is calculated to be approximately 3% of that in the absence of the amino acids, suggesting that LAT1-mediated transport from systemic circulation is saturated by endogenous LAT1 substrate amino acids. Therefore, pregabalin transport via LAT1 could be affected by changes in plasma amino acid levels. Indeed, it has been demonstrated by positron emission tomography that the uptake of L-[^18^F]fluoro-dopa, a substrate of LAT1, into human brain was inhibited to one-thirds during intravenous amino acids loading (42), at which the transport activity of LAT1 is also calculated from 3% to 1% by the amino acids loading. Likewise, the transport activity of LAT1 is calculated from 3% to 2% due to the increasing of the plasma amino acid concentrations by the intake of a protein rich meal (41), implying that the pregabalin influx via LAT1 at the BBB could be affected by the dietary protein intake.

LAT1 could also play an important role in pregabalin distribution to other organs. LAT1 is well known to be expressed in specific organs: brain, retina, spleen, placenta, spinal cord and bone marrow (13,43,44). One clinical action of pregabalin is suppression of neuropathic pain, which is caused by enhanced excitability in response to activation of synaptic inputs in the dorsal spinal cord. Pregabalin is considered to suppress dorsal horn excitability, but the mechanism of drug transfer across the blood-spinal cord barrier formed by capillary endothelial cells is still unclear. Notably, a recent investigation has demonstrated that pregabalin administration carries an increased risk of birth defects (45), which might imply transfer of pregabalin across the placental barrier. LAT1 protein has been immunohistochemically detected in microvessels of the spinal cord (13) and in human term placental syncytiotrophoblasts forming the placental barrier (46). Therefore, LAT1 might be involved in pregabalin distribution to the spinal cord across the blood-spinal cord barrier and to the fetus across the placental barrier.

## Conclusion

In this study, we have confirmed that LAT1 accepts pregabalin as a substrate, and contributes to uptake pregabalin in hCMEC/D3 cells. These findings suggest that LAT1 regulates the plasma membrane transport of pregabalin at the BBB. This has important implications for pregabalin pharmacokinetics, especially distribution to tissues.

## Acknowledgements and Disclosures

We thank Dr. Pierre-Olivier Couraud (Institut Cochin, Paris, France) for supplying hCMEC/D3 cells under license from INSERM. This study was supported in part by JSPS KAKENHI [Grants 15 K15007, 15 K08595, and 16 K08381]. It was also partially funded by Keio Gijuku Academic Development Funds, Keio University Doctorate Student Grant-in-Aid Program, The Mochida Memorial Foundation for Medical and Pharmaceutical Research, and The Uehara Memorial Foundation. This work was supported in part by MEXT-Supported Program for the Strategic Research Foundation at Private Universities.

## Electronic supplementary material

ESM 1 (DOCX 25 kb)

## Abbreviations

BBB: Blood-brain barrier
BCH: 2-Aminobicyclo-(2,2,1)-heptane-2-carboxylic acid
GABA: γ-Aminobutyric acid
hCMEC/D3 cells: Human immortalized brain endothelial cell line
HEK293 cells: Human embryonic kidney 293 cells
LAT: L-type amino acid transporter
MeAIB: α-Methylaminoisobutyric acid
OCTN: Organic cation transporter novel type
PAH: *p*-Aminohippuric acid
TEA: Tetraethylammonium
