# Supplementary material for: Transport of Pregabalin Via L-Type Amino Acid Transporter 1 (SLC7A5) in Human Brain Capillary Endothelial Cell Line
Source: Pharm Res. 2018 Oct 29;35(12):246. doi: 10.1007/s11095-018-2532-0 (PMC6208607; doi:10.1007/s11095-018-2532-0)
Supplement: Supplementary file 1 — (DOCX 25 kb) [file 11095_2018_2532_MOESM1_ESM.docx]

**Supplemental data**

**Supplemental Table 1 Effect of overexpression of sodium-coupled neutral amino acid transporters (SNATs) on cellular uptake of pregabalin.**

|  | Pregabalin uptake (µL/mg-protein) | | | | | | | |
| --- | --- | --- | --- | --- | --- | --- | --- | --- |
|  | Mock-transfected cells | | |  | SNATs-overexpressing cells | | | |
| SNAT1 | 15.8 | ± | 2.1 |  | 16.2 | ± | 3.5 |  |
| SNAT2 | 25.1 | ± | 8.7 |  | 25.3 | ± | 3.3 |  |
| SNAT4 | 28.3 | ± | 1.1 |  | 28.1 | ± | 1.1 |  |

SNAT1-, SNAT2-, SNAT4- and mock-transfected cells were incubated with pregabalin (10 µM) at 37^o^C for 5 min. Each value represents the mean ± SEM (n = 3-6).
